# The RTF-Compass: Navigating the Trade-Off Between Thermogenic Potential and Ferroptotic Stress in Adipocytes

**DOI:** 10.3390/cells15020170

**Published:** 2026-01-16

**Authors:** Minghao Fu, Manish Kumar Singh, Jyotsna Suresh Ranbhise, Kyung-Sik Yoon, Sung Soo Kim, Joohun Ha, Insug Kang, Suk Chon, Wonchae Choe

**Affiliations:** 1 Department of Biochemistry and Molecular Biology, School of Medicine, Kyung Hee University, Seoul 02447, Republic of Korea; andrewfupg@khu.ac.kr (M.F.);; 2 Department of Biomedical Science, Graduate School, Kyung Hee University, Seoul 02447, Republic of Korea; 3 Biomedical Science Institute, Kyung Hee University, Seoul 02447, Republic of Korea; 4 Department of Endocrinology and Metabolism, School of Medicine, Kyung Hee University, Seoul 02447, Republic of Korea

**Keywords:** adipose tissue thermogenesis, beige adipocytes, brown adipose tissue, ferroptosis, HIF-1α, iron metabolism, lipid peroxidation, NRF2–KEAP1 pathway, precision redox medicine, obesity

## Abstract

Adipose tissue thermogenesis is a promising strategy to counter obesity and metabolic disease, but sustained activation of thermogenic adipocytes elevates oxidative and lipid-peroxidation stress, increasing susceptibility to ferroptotic cell death. Existing models often treat redox buffering, hypoxia signaling and ferroptosis as separate processes, which cannot explain why similar interventions—such as antioxidants, β-adrenergic agonists or iron modulators—alternately enhance thermogenesis or precipitate tissue failure. Here, we propose the Redox–Thermogenesis–Ferroptosis Compass (RTF-Compass) as a framework that maps adipose depots within a space defined by ferroptosis resistance capacity (FRC), ferroptosis signaling intensity (FSI) and HIF-1α-dependent hypoxic tone. Within this space, thermogenic output follows a hormetic, inverted-U trajectory, with a Thermogenic Ferroptosis Window (TFW) bounded by two failure states: a Reductive-Blunted state with excessive antioxidant buffering and weak signaling, and a Cytotoxic state with high ferroptotic pressure and inadequate defense. We use this model to reinterpret genetic, nutritional and pharmacological studies as state-dependent vectors that move depots through FRC–FSI–HIF space and to outline principles for precision redox medicine. Although the TFW is represented as coordinates in FRC–FSI–HIF space, we use ‘Compass’ to denote a coordinate framework in which perturbations act as vectors that orient depots toward thermogenic or cytotoxic outcomes. Finally, we highlight priorities for testing the model in vivo, including defining lipid species that encode ferroptotic tone, resolving spatial heterogeneity within depots and determining how metabolic memory constrains reversibility of pathological states.

## 1. Introduction

Adipose tissue thermogenesis (ATT), mediated by brown and beige adipocytes, represents a pivotal metabolic target for combating the global burden of obesity and its associated dysfunctions [1,2,3]. While dissipating chemical energy as heat offers a tractable strategy to elevate whole-body energy expenditure, this metabolic benefit imposes a significant physiological cost: the activation of thermogenic programs compels adipocytes to operate at the precipice of cellular stress [2,4,5]. This creates a fundamental trade-off where the advantages of enhanced caloric burning are counterbalanced by the risks of sustained oxidative load, fluctuating oxygen supply, and lipid peroxidation, all of which compromise cell viability.

Under these high-flux conditions, adipocyte fate is governed by the dynamic interplay of three molecular pathways: NRF2, the primary antioxidant rheostat; HIF-1α, a metabolic brake induced by local hypoxia; and ferroptosis, an iron-dependent form of regulated cell death driven by lipid peroxides [6,7,8]. Importantly, redox-dependent modulation of thermogenesis presupposes adequate mitochondrial throughput (PDH–TCA–ETC flux); when oxidative metabolism is constrained, shifting antioxidant/pro-oxidant tone alone is unlikely to restore heat production. Accordingly, the RTF-Compass is intended to guide state-dependent combination strategies that pair flux-enabling stimuli with redox/ferroptosis/hypoxia tuning to keep depots within a safe thermogenic operating window [6,9,10]. Although these pathways are individually critical, current paradigms largely compartmentalize them, failing to explain why similar redox or hypoxic perturbations yield contradictory outcomes—either improving metabolic health or precipitating tissue failure [11,12,13].

To resolve these paradoxes and optimize ATT for obesity management, we propose the RTF-Compass. This hypothesis-driven framework integrates redox tone, thermogenic output, and ferroptotic signaling into a unified regulatory architecture. Central to this concept is the Thermogenic Ferroptosis Window (TFW), which posits that metabolic health is maintained not by eliminating stress, but by balancing it. Specifically, we argue that effective thermogenesis requires pro-oxidant stimuli (Ferroptosis signaling intensity, FSI; operationalized here as an index) to be sufficiently elevated to engage UCP1 (Uncoupling Protein 1) yet proportionately counterbalanced by antioxidant defenses (Ferroptosis Resistance Capacity, FRC) to prevent cytotoxicity.

This review synthesizes existing literature to formalize the RTF-Compass, demonstrating how this system distinguishes between “reductive-blunted,” “inert,” and “cytotoxic” failure modes. By translating qualitative observations into a rational decision model, we aim to provide de-risked translational strategies—from NRF2 modulation to dietary lipid interventions—that steer adipocytes toward a safe and sustainable thermogenic phenotype.

## 2. The Redox Axis: NRF2 and the Threshold of Reductive Blunting

Thermogenic adipocytes operate within a distinct metabolic paradigm where sustained oxidative load is not merely a respiratory byproduct, but a requisite signal for physiological function [9,14,15]. Stimuli such as cold exposure or β-adrenergic activation accelerate mitochondrial respiration and UCP1-mediated proton leak [2,16,17]. This metabolic surge drives rapid electron flux through the transport chain, often elevating reactive oxygen species (ROS) generation. ROS that promote versus suppress thermogenesis can arise from overlapping, but not identical, subcellular sources (mitochondrial and extra-mitochondrial sources). However, in the RTF-Compass, the complex crosstalk between mitochondrial and extra-mitochondrial redox compartments is not modeled separately; instead, it is integrated into a single axis, the ferroptosis signaling intensity (FSI), which reflects the net lipid-peroxidation-encoded output of these processes. Thus, the cellular redox architecture faces a critical discriminatory challenge: it must permit sufficient ROS accumulation to transduce thermogenic signals while preventing the transition into pathological oxidative stress.

### 2.1. The Molecular Rheostat: KEAP1–NRF2 Sensing

**Figure 1:**
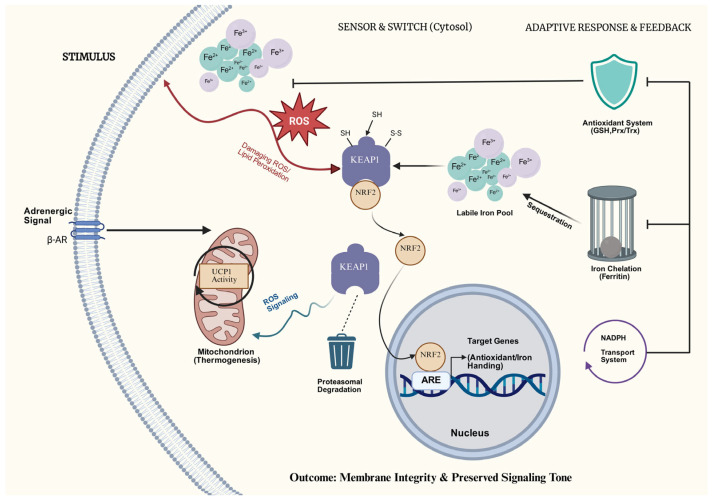
NRF2-mediated feedback control of redox signaling during thermogenic activation. β-adrenergic stimulation enhances mitochondrial respiration and UCP1-dependent thermogenesis, increasing reactive oxygen species (ROS) and labile iron that support redox signaling but, when excessive, threaten membrane integrity. Oxidative modification of KEAP1 cysteines stabilizes NRF2, promoting its nuclear translocation and binding to antioxidant response elements (AREs). NRF2 activation induces genes involved in glutathione biosynthesis, ferritin-mediated iron sequestration, and NADPH regeneration, thereby limiting lipid peroxidation and iron toxicity while preserving ROS-dependent signals required for sustained thermogenic function.

The KEAP1–NRF2 axis functions as the primary adaptive rheostat responsible for calibrating this delicate redox equilibrium [16,18,19]. Under basal conditions, NRF2 is constitutively repressed; it is physically sequestered by KEAP1 and targeted for ubiquitin-proteasomal degradation [20,21,22]. However, oxidative or electrophilic stress induces specific modifications of KEAP1 cysteine residues, which halts this degradation machinery [23,24]. This results in the stabilization and subsequent nuclear translocation of NRF2. As delineated in Figure 1, this nuclear accumulation orchestrates a coordinated cytoprotective program.

#### Integrated Antioxidant Defense and Iron Homeostasis

Upon activation, NRF2 drives a comprehensive transcriptional program that augments glutathione biosynthesis and turnover, reinforces peroxiredoxin/thioredoxin networks, and sustains NADPH regeneration via multiple metabolic shuttles [18,25,26]. Simultaneously, NRF2 exerts tight control over intracellular iron dynamics by upregulating machinery for iron sequestration and export, thereby limiting the propagation of lipid peroxidation [7,27,28]. Collectively, these mechanisms fortify adipocytes against oxidative insults during the high-flux state of thermogenesis, effectively elevating their FRC. Crucially, this adaptive response does not function as a binary switch that indiscriminately extinguishes ROS. Instead, NRF2 sculpts a permissive “redox tone”: a state where membrane integrity and respiratory control are preserved, yet a physiological pool of ROS remains available to transduce signals governing UCP1 activation and mitochondrial remodeling [29,30,31].

### 2.2. Context-Dependent Metabolic Outcomes

A pervasive paradox in contemporary literature is the observation that both NRF2 activation and its inhibition have been linked to a lean phenotype and enhanced energy expenditure [32,33]. We propose that these ostensibly conflicting findings do not represent a contradiction, but rather underscore NRF2’s function as a graded metabolic rheostat. Its impact is likely governed by specific cellular thresholds and distinct depot-specific microenvironments.

#### 2.2.1. Adaptive Activation in High-Stress Depots

In adipocyte depots inherently vulnerable to oxidative stress—specifically those burdened by high fatty acid flux or compromised vascular perfusion—a moderate upregulation of NRF2 activity confers a critical survival advantage [31,34,35]. By mitigating peroxidative damage and preserving mitochondrial integrity, NRF2 activation in this context expands the cell’s oxidative capacity. This adaptive response effectively safeguards against lipotoxicity, permitting sustained metabolic throughput without precipitating cell death [27,36,37].

#### 2.2.2. The Phenomenon of “Reductive Blunting”

Conversely, in environments characterized by low basal stress or high reductive reserve, supraphysiological NRF2 activity becomes maladaptive [36,38,39]. We define this pathological state as “Reductive Blunting.” Here, excessive NRF2 output indiscriminately scavenges the specific ROS and lipid-peroxide species required to drive thermogenic signaling cascades, effectively dampening pathways that converge on UCP1 and PGC-1α. The outcome is a distinct cellular phenotype: adipocytes that remain structurally viable yet are rendered metabolically inert due to “signal sterilization” [14,38,40].

### 2.3. The Thermogenic Ferroptosis Window (TFW) and the RTF-Compass

The RTF-Compass formalizes these complex dynamics by introducing two distinct quantitative metrics:

#### 2.3.1. Ferroptosis Resistance Capacity (FRC)

The integrated capability of the adipocyte to detoxify lipid hydroperoxides and buffer iron-driven chain reactions, a parameter largely governed by NRF2 activity [27,41,42].

#### 2.3.2. Ferroptosis Signaling Index (FSI)

The magnitude of lipid-peroxide-encoded signals generated during thermogenic stimulation [30,43,44]. FSI is not synonymous with general oxidative stress: here, it implies specifically the iron-coupled phospholipid-peroxidation tone that encodes both thermogenic signaling and ferroptotic liability [8,45]. In contrast, oxidative stress encompasses a broader spectrum of redox imbalance that may impair thermogenesis or trigger non-ferroptotic injury, even in the absence of iron-dependent lipid peroxidation as a dominant driver [46,47].

**Figure 2:**
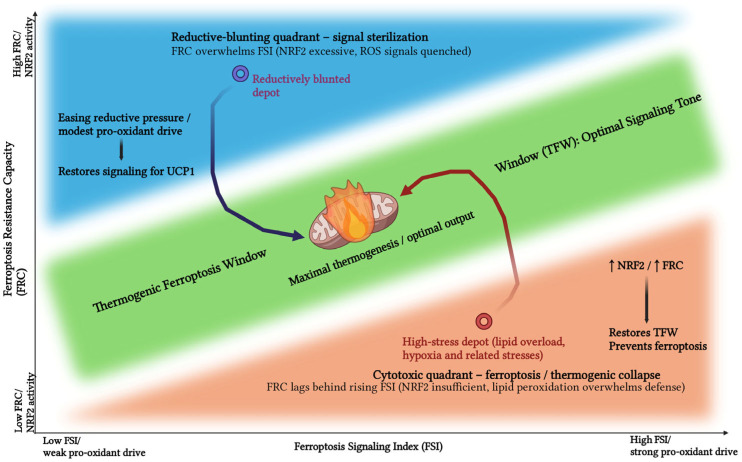
Schematic of the Thermogenic Ferroptosis Window (TFW) in the FRC–FSI plane. The green band marks the range in which ferroptosis signaling intensity (FSI, *x*-axis) is sufficient to drive thermogenic remodeling while ferroptosis resistance capacity (FRC, *y*-axis) still prevents membrane failure, yielding maximal thermogenic output. Depots displaced above this window (blue, reductive-blunting quadrant) have excessive NRF2/FRC that quenches ROS-encoded signals and suppresses UCP1 induction, whereas depots below it (orange, cytotoxic quadrant) exhibit low FRC relative to FSI, resulting in lipid-peroxidation-driven ferroptotic collapse. Arrows illustrate therapeutic trajectories that either ease reductive pressure or selectively boost NRF2/FRC to return depots into the TFW.

As illustrated in Figure 2, we postulate that thermogenic efficacy follows a hormetic, inverted-U trajectory defined by the interplay of these indices. Maximal thermogenesis is achieved exclusively within the Thermogenic Ferroptosis Window (TFW)—an optimal zone where FRC is sufficient to counterbalance cytotoxicity but permissible enough to preserve FSI. Deviation from this equilibrium precipitates two distinct failure modes:

Below the TFW: Insufficient NRF2 activity causes FRC to lag behind rising FSI, driving cells into a “Cytotoxic Quadrant” characterized by membrane compromise and thermogenic collapse [48,49].

Above the TFW: Supraphysiological NRF2 activity causes FRC to overwhelm FSI, resulting in “Reductive Blunting,” where the signal transduction requisite for UCP1 recruitment is effectively sterilized [50,51].

This framework unifies ostensibly conflicting literature by demonstrating that in high-stress depots, elevating NRF2 restores the TFW by preventing cell death, whereas in blunted depots, alleviating reductive pressure restores the oxidative cues essential for heat production [52,53,54,55].

#### 2.3.3. The Reductive Blunted State (High FRC, Low FSI)

This state emerges when antioxidant capacity disproportionately outweighs oxidative demand. Whether driven by constitutive NRF2 hyperactivation, excess reducing equivalents, or broad-spectrum antioxidant supplementation, lipid peroxides are scavenged prematurely [27,38,56]. Consequently, the adipocyte becomes refractory to oxidative signaling cues. Initiating chemical signals fail to surpass the sensing threshold, causing thermogenic pathways to remain dormant despite the presence of β-adrenergic stimuli [29,57]. This mechanism provides a mechanistic rationale for why blanket antioxidant regimens can paradoxically attenuate energy expenditure in otherwise responsive tissues.

#### 2.3.4. The Cytotoxic State (Low FRC, High FSI)

When ferroptotic pressure overwhelms resistance capacity, structural integrity is forfeited. Conditions such as severe mitochondrial uncoupling, toxic exposures, or glutathione depletion precipitate membrane rupture, organelle dysfunction, and overt ferroptotic cell death [54,58,59]. In this pathological mode, ferroptosis shifts from a signaling modality to a terminal executioner, effectively abolishing thermogenic output across the depot.

#### 2.3.5. The Quiescent State (Baseline FRC, Low FSI)

In the absence of cold stress or β-adrenergic drive, both signaling inputs and defense mechanisms operate at a homeostatic nadir. The depot functions primarily as a storage reservoir, maintaining structural stability without significant heat production [29,38,60]. This state is characterized by low FSI and basal, constitutive levels of FRC.

## 3. HIF-1α as an Oxygen-Sensing Rheostat in Thermogenic Adipose Tissue

Thermogenic adipocytes function within a dynamic landscape of oscillating oxygen tension. During acute cold exposure, brown adipose tissue (BAT) rapidly accelerates mitochondrial respiration and UCP1-mediated proton leak, drastically increasing oxygen consumption and generating a state of “physiological hypoxia [61,62,63].” Conversely, in obesity, the expansion of white adipose tissue (WAT) frequently outstrips its vascular supply, resulting in persistent, pathological hypoxia. The master regulator of this hypoxic response is HIF-1α (Hypoxia-Inducible Factor alpha) [35,64,65]. Under oxygen-limiting conditions, prolyl hydroxylase (PHD) activity is inhibited, leading to HIF-1α stabilization, dimerization with HIF-1β, and the subsequent activation of transcriptional programs governing glycolysis, angiogenesis, and stress survival [66,67].

### 3.1. Adaptive Hypoxia

The Transient Pulse: In BAT, a transient pulse of HIF-1α signaling appears to be an adaptive requisite for thermogenesis [68,69]. Reactive oxygen species (ROS) generated during UCP1 activation can further stabilize HIF-1α, independent of hypoxia alone. The resulting transcriptional cascade upregulates glucose transporters (GLUTs) and glycolytic enzymes to fuel the rapid metabolic demand, enhances lactate handling, and promotes VEGF-driven vascular remodeling to improve perfusion [70,71,72]. Loss-of-function studies corroborate this adaptive role: selective depletion of HIF-1α in brown adipocytes impairs glucose utilization and blunts maximal thermogenic respiration, indicating that acute hypoxia signaling is essential to sustain substrate flux when oxygen availability becomes the rate-limiting step [6,68].

### 3.2. Maladaptive Hypoxia

The Chronic Brake: Pathological consequences emerge when HIF-1α activity becomes excessive or chronically sustained [64,73]. In obese WAT, chronic hypoxia correlates with pro-inflammatory signaling and insulin resistance, reflecting a maladaptive shift toward glycolytic reliance and extracellular matrix fibrosis [72,74,75]. Genetic models of constitutive HIF-1α activation recapitulate this phenotype: mice with sustained HIF-1α signaling in adipose tissue exhibit suppressed BAT oxygen consumption, reduced mitochondrial density, and downregulated thermogenic regulators [63,68,76]. This culminates in diminished whole-body energy expenditure and increased adiposity, particularly under cold stress. Mechanistically, chronic HIF-1α drives a metabolic rerouting—primarily by upregulating Pyruvate Dehydrogenase Kinases (PDKs) to inhibit Pyruvate Dehydrogenase (PDH) [6,69,77]. This “Warburg-like” switch shunts metabolism toward glycolysis and lactate production while throttling tricarboxylic acid (TCA) cycle flux and electron transport. The result is a “Protected but Low-Throughput” state: cells remain viable via glycolysis, but mitochondrial biogenesis, fatty acid oxidation, and UCP1-dependent uncoupling are actively repressed. This chronic program stands in sharp contrast to the transient, thermogenesis-supportive HIF-1α pulse seen in BAT [6,68,78]. A direct comparison of these opposing modes is summarized in Table 1.

### 3.3. Integrating HIF-1α into the RTF-Compass

The RTF-Compass integrates HIF-1α alongside redox control (NRF2) as a second, orthogonal module governing thermogenic efficacy. Within this framework, hypoxia signaling must be “calibrated” to thermogenic demand: insufficient HIF-1α during activation starves the system of necessary glycolytic fuel and angiogenic support, whereas excessive HIF-1α constricts mitochondrial throughput and silences UCP1 programs [90,91,92]. Practically, BAT performance follows an inverted-U relationship with HIF-1α tone, analogous to the TFW defined for NRF2.

Optimal Zone: Transient, moderate HIF-1α activation maintains ATP homeostasis, preserves ratios, and enhances vascular perfusion, allowing ROS-encoded cues to propagate thermogenic gene expression [63,93].

Maladaptive Zone: Chronic activation shifts the metabolic setpoint toward glycolytic conservation, effectively acting as a metabolic brake. Therefore, therapeutic strategies must align HIF-1α amplitude and duration with thermogenic workload [82,94,95]. This entails pairing brief, hypoxia-supportive cues (coinciding with peak oxygen demand) with safeguards against chronic stabilization, such as enhancing vascular delivery. By monitoring biomarkers of this window—including GLUT expression, PDK1 levels, lactate accumulation, and VEGF induction—the RTF-Compass aims to steer adipose tissue away from both “oxidative failure” and “glycolytic stagnation,” securing a safe and productive thermogenic operating zone [78,96].

Cancer-associated adipose remodeling provides an additional disease context in which HIF-1α tone can be coupled to UCP1 induction yet decoupled from beneficial thermogenic output. Recent studies indicate that tumor-adjacent WAT can acquire beige/brown features (e.g., increased UCP1 and mitochondrial markers) in settings such as breast cancer xenografts and human renal cancer peritumoral fat. In these niches, tumor-driven metabolic pressure favors increased GLUT expression and glycolytic flux, with elevated lactate production/handling that can further reinforce HIF-1α programs and VEGF-linked angiogenic signaling, thereby potentially supporting tumor progression (e.g., via vascular remodeling and pro-growth stromal cues) [97,98,99]. Within the RTF-Compass, these observations can be conceptualized as a context-dependent vector that elevates hypoxic tone and glycolytic dependence, illustrating why “browning” markers alone may not equate to a net thermogenic state and why calibration of HIF-1α amplitude/duration remains central across distinct pathophysiological environments.

## 4. Ferroptotic Lipid Signaling as a Metabolic Checkpoint for Adipose Thermogenesis

Thermogenic adipocytes must simultaneously generate heat and preserve membrane integrity in an environment rich in reactive oxygen species and redox-active iron. In the preceding sections, we focused on NRF2-dependent antioxidant defense and HIF-1α–mediated hypoxic responses as key determinants of this balance [100,101]. However, neither axis alone explains how oxidative and iron-dependent stress is converted into a graded signal that can promote thermogenic remodeling or, when uncontrolled, trigger cell death. Ferroptosis, an iron-dependent form of regulated necrosis driven by phospholipid peroxidation, provides a mechanistic link between these processes [100,101]. In this section, we consider ferroptotic lipid signaling as a metabolic checkpoint for adipose thermogenesis, introduce the concepts of ferroptosis resistance capacity (FRC) and ferroptosis signaling intensity (FSI), and develop the idea of a Thermogenic Ferroptosis Window (TFW) that formalizes how thermogenic efficacy emerges from the interplay between these parameters.

### 4.1. The Precipice of Metabolic Stress

Thermogenic adipocytes operate at the physiological limit of cellular stress. The rapid mitochondrial electron flux and continuous membrane remodeling required for heat generation inherently prime the cellular environment for lipid peroxidation [102]. Ferroptosis, an iron-dependent, non-apoptotic form of regulated cell death, ensues when the accumulation of phospholipid hydroperoxides surpasses the cell’s detoxification capacity [8,41,103]. Under homeostatic conditions, Glutathione Peroxidase 4 (GPX4) efficiently reduces these toxic hydroperoxides to benign alcohols. However, during peak thermogenic drive—triggered by acute cold exposure or β-adrenergic stimulation—this defensive system can be compromised due to GPX4 downregulation or glutathione depletion [38,49,104]. The consequence is an escalation of “ferroptotic pressure,” often evidenced by elevated markers such as malondialdehyde (MDA). If this pressure exceeds the cellular threshold, unmitigated peroxidation disrupts mitochondrial integrity and UCP1 machinery, precipitating a collapse in thermogenic output [55,105,106].

### 4.2. Hormesis: The Instructional Value of Oxidative Tension

Crucially, however, this oxidative tension carries specific instructional value when maintained below lethal thresholds. Emerging evidence supports the view that thermogenesis operates as a hormetic process: sub-lethal ferroptotic stress is not merely a toxic byproduct, but a requisite signal for adaptive remodeling [53,55,107]. Controlled bursts of lipid peroxides and their aldehyde derivatives act as local signaling cues, triggering stress-responsive transcription factors that prime thermogenic gene programs. At sublethal levels, candidate ‘instructional’ signals likely include electrophilic lipid aldehydes such as 4-hydroxynonenal (4-HNE), which can function as a second messenger via selective protein adduction and has been shown to modulate uncoupling-protein-dependent proton conductance [108,109]. A second candidate class is oxidized phospholipids (OxPLs) generated through the ACSL4–LPCAT–lipoxygenase axis; notably, specific oxidized phosphatidylethanolamines are considered canonical lipid mediators in ferroptosis and could plausibly encode graded ‘tone’ rather than binary damage [110,111,112]. In parallel, cold-activated adipose can release lipoxygenase-derived oxylipins with endocrine activity (e.g., 12-HEPE), supporting the broader concept that oxidized lipid species can transmit adaptive metabolic instructions [113,114,115]. The physiological challenge, therefore, lies in capturing this signaling utility without crossing the threshold into cell death [12,116].

### 4.3. NRF2: The Arbiter of the Ferroptosis Window

NRF2 functions as the central arbiter of this delicate balance. By governing the transcriptional expression of glutathione-biosynthetic enzymes, GPX4, and iron-sequestering proteins like ferritin, NRF2 dictates the FRC—defined as the cell’s integrated potential to neutralize lipid toxicity and restrain labile iron [27,117,118]. Importantly, a robust FRC does not eliminate lipid-peroxide signaling entirely; rather, it shapes the Ferroptosis Signaling Index (FSI), which represents the effective load of pro-oxidant lipids that remain available to communicate with thermogenic circuits [119,120,121].

### 4.4. The Thermogenic Ferroptosis Window (TFW)

The interaction between FRC and FSI delineates a Thermogenic Ferroptosis Window (TFW) in which thermogenic efficacy follows an inverted-U trajectory: function is maximal only when FRC is sufficient to prevent catastrophic ferroptosis yet permissive enough to allow essential peroxide-based signaling. When FRC lags behind rising FSI—for example, during intense cold stress or glutathione depletion—adipocytes enter a Cytotoxic state, characterized by progressive membrane damage, loss of UCP1 function, and collapse of thermogenic output [30,122,123]. Conversely, when FRC is excessively high, such as under chronic NRF2 hyperactivation or prolonged antioxidant exposure, lipid-peroxide tone is over-quenched; cells remain viable but adopt a Reductive-Blunted state in which beige recruitment and sustained UCP1 activity are attenuated because stress-encoded signals are effectively sterilized [29,57].

### 4.5. Therapeutic Implications

This framework provides a rationale for precision therapeutics. In oxidatively stressed or degenerative adipose depots, strategies that bolster FRC—such as enhancing GPX4 function or chelating labile iron—may preserve thermogenic viability. In contrast, in metabolically inactive or reductively biased depots, therapeutic easing of FRC may be necessary to restore the oxidative tone required for beigeing and optimal UCP1 function.

## 5. Integrating the Hypoxic Axis: HIF-1α as a Dynamic Modulator of the TFW

Section 4 established that adipose thermogenesis is constrained by a ferroptotic safety margin: productive thermogenic remodeling is possible only when lipid-peroxide–encoded signals are sufficiently strong to drive adaptation, yet sufficiently buffered to avoid membrane failure. Building on this logic, the RTF-Compass incorporates hypoxic pressure as a third axis that explains why apparently similar thermogenic stimuli can yield opposite outcomes across depots and disease contexts. Importantly, the hypoxic response governed by HIF-1α should not be treated as a binary “on/off” brake [61,124]. Instead, the Compass frames HIF-1α as a temporally governed modulator that reshapes the accessibility and stability of the Thermogenic Ferroptosis Window (TFW) across time.

The practical implication is that oxygen availability and perfusion are not passive background variables. Thermogenic activation increases oxygen demand and can generate a physiological hypoxic burden even in otherwise healthy tissue; conversely, obesity, hypertrophy, fibrosis, and inflammation can impose persistent vascular insufficiency and diffusion limitations [61,125]. These settings differ not only in magnitude but in duration, and duration is decisive: a hypoxic signal that resolves can support adaptation, whereas a hypoxic signal that persists tends to constrain metabolic flexibility, bias inflammatory recruitment, and destabilize thermogenic competence [13,78]. Within the RTF-Compass, HIF-1α therefore functions as an orthogonal axis that changes the shape of the operating landscape rather than redefining its core redox–ferroptosis balance.

### 5.1. Hypoxia-Dependent Modulation of Operational States

With the redox and ferroptosis axes defined earlier, adipose depots can be organized into a small number of recurring operational states, but their stability depends on hypoxic tone. In the TFW, thermogenic signaling is productive because oxidative and lipid-peroxidation cues remain within a tolerable range and are met by adequate protective capacity [38,49,126]. Under these conditions, a depot can sustain high mitochondrial throughput and thermogenic effector programs without accumulating irreversible lipid damage. Critically, the hypoxic response in this regime is typically transient and resolving: short-lived HIF-1α activity can promote compensatory processes such as glycolytic support, vascular remodeling, and nutrient delivery, which help the tissue absorb the energetic and redox demands of thermogenesis [70,127,128].

Deviation from this optimal regime yields principled failure modes that are strongly influenced by hypoxic dynamics. In a Reductive-Blunted state, defensive capacity is disproportionately high relative to signaling strength. Even if adipocytes remain viable, excessive quenching of stress-encoded cues can sterilize the very lipid-peroxide signals needed for recruitment and maintenance of thermogenic machinery [40,57,129]. In this setting, increasing stimulation may not restore thermogenesis if the depot is “signal-poor,” and the tissue may preferentially store energy rather than dissipate it [57,129,130,131]. At the opposite extreme, the Cytotoxic state reflects a depot in which signaling and damage are no longer separable: lipid peroxidation overwhelms detoxification and repair, and attempts to further amplify thermogenesis primarily accelerate injury rather than improve energy expenditure [122,132,133].

**Figure 3:**
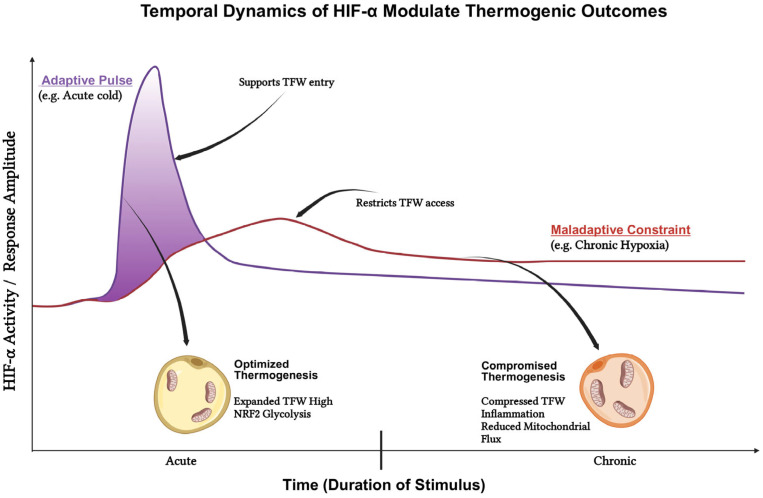
Temporal dynamics of HIF-1α signaling reshape the operational boundaries of the Thermogenic Ferroptosis Window (TFW). This schematic illustrates how the duration and intensity of hypoxic stress function as a dynamic axis modulating adipocyte thermogenic outcomes within the RTF-Compass framework. Transient HIF-1α activation (purple curve), characteristic of acute cold exposure, generates a short-lived hypoxic pulse that enhances glycolytic flux, vascular remodeling, and nutrient delivery. These adaptive responses expand the Thermogenic Ferroptosis Window (TFW), promoting optimized thermogenesis supported by high Ferroptosis Resistance Capacity (FRC) and controlled Ferroptosis Signaling Index (FSI). In contrast, chronic HIF-1α stabilization (red curve), as seen during prolonged hypoxia, imposes a maladaptive constraint that restricts TFW access. Sustained hypoxia suppresses mitochondrial flux, elevates inflammatory tone, and reduces metabolic flexibility, thereby narrowing the safety margin of the FRC–FSI equilibrium and predisposing adipocytes to thermogenic failure. The figure highlights these divergent trajectories across time, emphasizing HIF-1α as a non-binary, temporally governed modulator of thermogenic resilience.

The hypoxic axis modulates how readily depots move between these regimes. Transient HIF-1α pulses—as may occur during acute cold exposure or intermittent metabolic challenges—can temporarily expand the effective TFW by improving perfusion and supporting controlled excursions toward higher signaling without catastrophic damage [68,95,134]. By contrast, chronic HIF-1α stabilization—as expected in hypertrophic, inflamed, or fibrotic depots—tends to compress the TFW. Persistent hypoxic pressure suppresses mitochondrial flux, elevates inflammatory tone, and reduces metabolic flexibility, thereby narrowing the safety margin within which signaling remains constructive [73,135,136]. Under these compressed conditions, even modest increases in lipid-peroxidation tone may precipitate failure rather than adaptation. Figure 3 schematizes this central idea by contrasting a resolving hypoxic “pulse” with a prolonged hypoxic “constraint,” and by illustrating how these distinct temporal profiles reshape access to safe thermogenesis.

### 5.2. Dynamic Trajectories on the RTF-Compass

While operational states provide a useful snapshot, adipose depots in vivo rarely remain fixed. Instead, they trace trajectories across the Compass as environmental cues, nutritional context, inflammation, and therapeutic exposures fluctuate [79,137,138,139]. This trajectory view is essential because the same intervention can be beneficial or harmful depending on a depot’s starting coordinates and on whether hypoxic tone resolves or persists. In this framework, interventions are best interpreted as vectors of motion in the combined space of protective capacity, signaling intensity, and hypoxic pressure, rather than as simple “activators” or “inhibitors” [125,137,140].

One class of trajectories can be described as adaptive pulses. Acute or intermittent challenges transiently elevate thermogenic flux and associated signaling while simultaneously inducing protective programs and a resolving hypoxic response [141,142,143]. When these axes remain coordinated, the depot moves into or within the TFW: signaling is heightened but buffered, perfusion improves, and thermogenic machinery is reinforced [114,144,145,146]. After the stimulus resolves, hypoxic tone and signaling intensity return toward baseline, whereas parts of the remodeled network and protective capacity may be retained, supporting durable thermogenic competence without cumulative tissue injury [35,147].

In contrast, maladaptive constraint trajectories arise when hypoxic and lipid-peroxidative stresses remain chronically elevated while protective capacity fails to keep pace [49,122,133]. Persistent overnutrition, unresolved inflammation, or sustained vascular rarefaction can drive a depot toward high hypoxic tone and heightened stress without permitting recovery [85,148,149,150]. In Compass terms, the TFW becomes compressed and the depot drifts toward a Cytotoxic regime in which additional stimulation predominantly accelerates damage [30,122,126,133]. The trajectory perspective therefore provides the conceptual bridge to Section 6: rational therapies should be designed and evaluated as state-dependent vectors that either re-expand the TFW (for constrained, high-risk depots) or avoid deepening signal deficiency (for Reductive-Blunted depots), rather than applying a one-size-fits-all thermogenic push.

## 6. Implications for Intervention Strategies: The RTF-Compass

The RTF-Compass framework argues that thermogenic efficacy and ferroptotic risk are determined not by single pathways in isolation, but by the joint configuration of ferroptosis signaling intensity (FSI), ferroptosis resistance capacity (FRC), and HIF-1α-dependent hypoxic tone. Building on this logic, interventions must be evaluated as state-dependent vectors that either expand or compress the Thermogenic Ferroptosis Window (TFW), rather than as uniformly beneficial or harmful stimuli [38,90,151]. A practical implication of this framework is that ‘Compass coordinates’ must be approximated before therapy selection. While depot biopsy would be the most direct approach, a realistic near-term path is to validate non-invasive proxy panels that reflect the FRC/FSI balance and hypoxic tone. For example, systemic lipid peroxidation readouts (e.g., F2-isoprostanes, MDA/4-HNE adduct burden) could be interpreted alongside antioxidant capacity (e.g., circulating/immune-cell GSH metrics) and iron-status indices (e.g., ferritin, transferrin saturation, hepcidin-related patterns) to estimate whether ferroptotic signaling outweighs buffering [152,153,154,155,156]. In parallel, imaging-based estimates of thermogenic engagement (e.g., FDG-PET/CT–based BAT activity) and oxygenation/perfusion surrogates could help contextualize whether HIF-1α tone is resolving (adaptive) or persistent (maladaptive). Although these measures are imperfect and not depot-specific, the Compass provides a testable hypothesis: biomarker ratios that track ‘signal-to-buffer’ should predict whether a thermogenic push will expand the TFW or precipitate cytotoxic failure [157,158,159].

Recent in vivo evidence illustrates this principle. Intermittent or time-restricted fasting promotes browning of white adipose tissue (WAT), limits fat deposition, and reduces inflammatory markers, partly through VEGF-mediated alternative activation of macrophages and improved vascular perfusion [160,161,162,163]. In RTF-Compass terms, these regimens modestly increase FSI while concurrently supporting FRC and alleviating local hypoxia, thereby nudging depots toward the TFW. By contrast, adipose-specific deletion of transferrin receptor 1 (TfR1) limits iron uptake and blunts BAT thermogenesis, reflecting insufficient ferroptotic tone [133,164,165]. Conversely, adipocyte-specific ACSL4 overexpression or ferritin heavy chain (FTH) loss protects against high-fat diet-induced adipose expansion by activating ferroptosis signaling in vivo—beneficial only when applied to depots with adequate FRC [30,166,167].

The lipid metabolite 5,15-dihydroxyeicosatetraenoic acid (5,15-DiHETE) offers a more explicit example of a Compass-guided intervention. By inducing ferroptosis and promoting degradation of hypoxia-inducible factor-1α (HIF-1α), 5,15-DiHETE relieves repression of a c-Myc–PGC-1β–driven thermogenic program [168,169,170]. This maneuver transiently elevates FSI while decompressing a hypoxia-compressed TFW, thereby enhancing thermogenic competence in depots that would otherwise remain constrained by chronic HIF-1α activity [68,171,172]. Collectively, these findings indicate that RTF-Compass-guided therapy requires estimating a depot’s “coordinates” (FRC/FSI balance and hypoxic tone) before intervention. To translate this framework beyond biopsy-based studies, candidate non-invasive surrogates—such as circulating lipid peroxidation products (e.g., MDA/4-HNE adduct burden) relative to systemic antioxidant capacity (e.g., GSH-related measures) and iron-handling markers (e.g., ferritin/transferrin indices)—should be validated as operational readouts of Compass state [173,174,175].

### 6.1. Context-Dependent Therapy

Adipose tissue maintains whole-body energy balance by coordinating lipid storage, mobilization, and thermogenesis. Canonical regulators such as PPARγ and PGC-1α exemplify how the same target can yield divergent outcomes depending on the depot’s initial position on the Compass. PPARγ agonism enhances adipogenesis and insulin sensitivity, but in Reductive-Blunted depots with already high FRC and low FSI, further reinforcement of storage programs may deepen metabolic inertia [176,177,178]. In contrast, in Cytotoxic depots where FRC is deficient, modest PPARγ activation can improve lipid handling and indirectly support FRC by stabilizing mitochondrial function. Similarly, PGC-1α–driven mitochondrial biogenesis and thermogenesis can either restore TFW access in quiescent or reductively blunted depots, or precipitate ferroptotic failure in depots already burdened by high FSI and chronic hypoxia [179,180,181,182].

Adipokines further modulate Compass coordinates at a systems level. Pro-inflammatory mediators such as TNF-α, MCP-1, and IL-6 amplify ROS production, destabilize iron handling, and promote chronic HIF-1α stabilization, thereby driving depots toward the Cytotoxic state [46,78,183,184]. In contrast, adipokines such as apelin or certain chemokines associated with browning support vascular remodeling, substrate delivery, and controlled thermogenic activation, facilitating safe excursions into the TFW [34,185,186,187].

Ferroptosis-regulating pathways sit at the core of this context dependence. As outlined earlier, ferroptotic signaling can promote thermogenic recruitment by inducing PHD2 and destabilizing HIF-1α, thereby activating the c-Myc–PGC-1β axis [188,189,190]. However, excessive or poorly buffered ferroptosis converts this instructional signal into terminal membrane failure. This inherent bidirectionality underscores the need for context-dependent, precision-based therapeutic strategies [191,192,193,194].

### 6.2. Specific Intervention Vectors

Within the RTF-Compass framework, concrete interventions can be interpreted as vectors that predominantly modulate FRC, FSI, and/or HIF-1α tone. Classical thermogenic stimuli such as cold exposure and pharmacological β3-adrenergic agonists (e.g., mirabegron) primarily raise FSI by increasing mitochondrial flux and lipolytic fatty acid supply [5,14,195]. In depots with sufficient FRC and transient HIF-1α responses, these vectors move the tissue into or within the TFW, enhancing thermogenesis and energy expenditure. However, in Cytotoxic depots characterized by low FRC and chronic hypoxia, the same interventions may accelerate ferroptotic injury and cardiovascular stress, necessitating careful dose optimization and patient selection [196,197,198].

Targeting ferroptosis machinery provides a second class of vectors. GPX4, a central regulator of ferroptosis, defines the upper limit of FRC; its loss or pharmacological inhibition (e.g., RSL3, erastin-mediated system x_c^−^ blockade) promotes ferroptotic signaling and has been implicated in BAT involution [199,200,201]. Such interventions may be useful for decompressing Reductive-Blunted depots by lowering excessive FRC and reinstating a permissive FSI, but they are hazardous in already Cytotoxic or hypoxia-compressed depots. Conversely, iron chelators (deferoxamine, ciclopirox) or lipid-peroxidation inhibitors (ferrostatin-1, liproxstatin-1) move depots downward along the FSI axis, reducing ferroptotic pressure but also narrowing TFW access if FSI becomes too low to sustain thermogenic signaling [117,194,202].

Additional regulatory layers operate through proteostasis and non-coding RNAs. Nfe2L1, a master regulator of proteasome function, is essential for BAT remodeling during cold adaptation; its loss promotes ferroptosis and destabilizes thermogenic balance. Long noncoding RNAs such as AATBC and LINC00473 enhance adipose browning and mitochondrial dynamics, effectively supporting safe transitions into the TFW [203,204]. In contrast, the lncRNA Lexis acts as a brake on canonical thermogenesis; genetic or pharmacological Lexis inhibition enhances ATP2A2-mediated UCP1-dependent and -independent thermogenesis and can be conceptualized as a vector that selectively increases FSI in depots with adequate FRC [166,205,206].

Taken together, these examples motivate a structured, Compass-based classification of interventions. Table 2 organizes representative agents by their primary target mechanism, predominant vector on the Compass (e.g., ↑ FRC, ↓ FSI, decompression of HIF-1α), and the depot state in which they are predicted to be most beneficial or harmful. In practical terms, Table 2 serves as a decision matrix for aligning specific pharmacological or lifestyle interventions with the underlying state of the depot.

### 6.3. Rescuing the Cytotoxic Depot

Among the states defined by the RTF-Compass, the Cytotoxic depot (low FRC, high FSI, often accompanied by chronic HIF-1α stabilization) represents a particularly vulnerable configuration. Thermogenic adipocytes accumulate iron and depend on high iron flux to sustain mitochondrial respiration, yet brown adipose tissue (BAT) has a more limited iron storage capacity than white adipose tissue (WAT) [170,217,218]. Adipose-specific impairment of transferrin receptor-mediated iron uptake reduces BAT thermogenic activity, whereas systemic iron chelation with agents such as deferoxamine or ciclopirox suppresses Fenton chemistry but also attenuates thermogenic function. These findings point to a narrow therapeutic margin: partial reduction in ferroptotic pressure can mitigate injury, but excessive suppression of iron-dependent redox signaling risks excluding the depot from the Thermogenic Ferroptosis Window (TFW) [30,133,219].

Within the RTF-Compass framework, restoration of redox buffering emerges as a key determinant of ferroptosis sensitivity in Cytotoxic depots. Strategies that replenish intracellular glutathione pools or enhance GPX4 activity expand FRC, re-balancing survival pathways against ferroptotic signaling and potentially allowing depots to re-approach the TFW [199,220,221]. At the same time, the model predicts that excessive reinforcement of FRC could shift the system toward a Reductive-Blunted state, in which thermogenic signals are dampened despite preserved cellular integrity. This highlights the importance of dose, timing, and depot-specific context when considering ferroptosis-modulating therapies [38,54,222,223].

Hypoxia adds an additional layer of complexity. Lactate produced by obese, hypoxic WAT can initially promote browning by inducing UCP1 and recruiting beige adipocytes, but persistent adipose hypoxia increases ROS production, impairs oxidative phosphorylation, and accelerates ferroptotic progression [126,224,225]. From an RTF-Compass perspective, interventions that alleviate chronic hypoxia—by improving angiogenesis, reducing fibrosis, or enhancing perfusion—would be expected to decompress the TFW and reduce the likelihood that further increases in FSI precipitate ferroptotic failure. Thus, rescuing the Cytotoxic depot is likely to require coordinated modulation of iron handling, antioxidant capacity, and hypoxic tone, and future studies will be needed to test whether such combined strategies can reliably restore thermogenic competence in vivo [126,226,227,228].

## 7. Future Directions and Outstanding Questions

The RTF-Compass offers an integrative conceptual framework linking redox control, hypoxia signaling, and ferroptotic lipid peroxidation in thermogenic adipose tissue, while also underscoring key evidence gaps that must be resolved for experimental and clinical translation. Three outstanding dimensions are particularly important. First, the field must define the specific lipid species—and corresponding biochemical pathways—that determine ferroptotic “signaling tone” under thermogenic stress. Second, it remains unclear how RTF-Compass coordinates are spatially distributed across adipose depots and within individual depots, including the extent of microenvironment-driven heterogeneity. Third, the mechanisms by which prior metabolic stress is temporally “remembered” through persistent remodeling of redox and ferroptosis networks require clarification. Addressing these questions will be essential to rigorously test, refine, and ultimately operationalize the RTF-Compass in vivo.

### 7.1. Decoding the Lipid Basis of Ferroptotic Signaling

Most studies of oxidative stress in adipose tissue still rely on bulk markers such as malondialdehyde, 4-hydroxynonenal, or generic ROS-sensitive probes. While useful as global readouts, these measures cannot distinguish between lipid oxidation events that promote adaptive thermogenic signaling and those that initiate ferroptotic death. Within the RTF-Compass framework, this distinction is central: the Ferroptosis Signaling Index (FSI) is intended to reflect the burden of specific lipid species that engage thermogenic or cytotoxic programs, not simply total oxidative damage.

Future work will therefore need to move toward high-resolution lipidomics capable of resolving oxidized phospholipid species at the level of headgroup, acyl chain composition, and subcellular localization. Examples include separating oxidized phosphatidylethanolamines, which are canonical mediators of ferroptosis, from oxidized cardiolipins associated with mitochondrial membrane injury, and linking these profiles to defined thermogenic outcomes. Combining targeted lipidomics with isotope tracing, organelle fractionation, and functional perturbation of lipid-metabolizing enzymes (e.g., ACSL4, LPCATs, lipoxygenases) should allow construction of a more quantitative map between individual lipid species and the FSI axis. Such data will be necessary to determine whether ferroptotic tone can be selectively tuned toward adaptive remodeling without triggering cell death.

### 7.2. Spatial Heterogeneity and “Micro-Compass” States

Adipose tissue is structurally and functionally heterogeneous, with steep gradients in oxygen tension, immune cell composition, and nutrient delivery across relatively small spatial scales. It is therefore unlikely that a single, uniform set of RTF-Compass coordinates applies to an entire depot. Rather, individual adipocytes are expected to occupy distinct microenvironments, with local differences in FRC, FSI, and HIF-1α tone. For example, cells adjacent to a well-perfused vessel may reside near the Thermogenic Ferroptosis Window, whereas cells in a hypoxic, fibrotic core may already be constrained toward Cytotoxic or Reductive-Blunted states.

Resolving this heterogeneity will require spatially resolved approaches. Spatial transcriptomics, imaging mass spectrometry, and in vivo redox or oxygen sensors could be used to define “micro-compass” states within specific niches, linking local gene expression, lipid oxidation, and hypoxia markers to thermogenic capacity. Integrating these data with three-dimensional maps of vascular and immune architecture may clarify whether effective browning requires global reprogramming of a depot or predominantly involves expansion of pre-existing permissive niches. This question has direct translational relevance, as interventions that are effective at the well-perfused periphery may be insufficient to correct dysfunction in poorly perfused regions.

### 7.3. Redox Hysteresis and Metabolic Memory

Clinical and experimental observations suggest that adipose tissue may retain a “memory” of prior metabolic stress. Even after weight loss or normalization of systemic metabolic parameters, thermogenic function and inflammatory tone do not always fully recover. Within the RTF-Compass framework, such phenomena can be viewed as a form of redox hysteresis, in which past insults shift the effective set-points of FRC, FSI, or HIF-1α signaling and restrict the ability of a depot to re-enter the Thermogenic Ferroptosis Window.

Potential mechanisms for this hysteresis include persistent iron accumulation, long-lived changes in immune cell composition, and epigenetic or transcriptional reprogramming of NRF2, HIF-1α, or ferroptosis regulators. Disentangling these contributions will require longitudinal models that track Compass coordinates over time, including during cycles of weight gain and loss, as well as after targeted perturbations of redox or iron-handling pathways. Combining time-resolved multi-omics with functional assessments of thermogenesis could reveal whether pathological Compass states are reversible or represent semi-stable “metabolic scars” that limit the efficacy of late interventions.

### 7.4. Concluding Remarks

Ferroptosis in adipose tissue exemplifies a broader principle in metabolic biology: the same oxidative and lipid-peroxidation processes that threaten cell viability can also serve as indispensable signals for adaptive remodeling. The RTF-Compass proposes one way to formalize this balance, positioning NRF2-dependent redox buffering, HIF-1α-mediated hypoxia responses, and ferroptotic lipid signaling within a single operating space that includes a Thermogenic Ferroptosis Window, a Reductive-Blunted state, and a Cytotoxic state. Rather than advocating for uniformly “pro-oxidant” or “antioxidant” strategies, this framework emphasizes the importance of matching intervention type and intensity to the underlying depot state.

Going forward, a key challenge will be to define measurable biomarkers and experimental readouts that approximate FRC, FSI, and hypoxic tone in vivo, and to determine how these parameters change across time, space, and disease context. As these tools are developed, the RTF-Compass may serve less as a fixed model and more as a guiding hypothesis for designing and interpreting studies of thermogenic adipose tissue. Ultimately, the goal is not to eliminate stress, but to maintain it within a controlled range that preserves structural integrity while enabling the thermogenic functions required for metabolic health.

## Figures and Tables

**Table 1:** Adaptive versus maladaptive modes of HIF-1α signaling in thermogenic adipose tissue.

Feature	Adaptive HIF-1α Response	Maladaptive HIF-1α Response	References
Trigger	Acute cold exposure; Transient β-adrenergic stimulation	Chronic Obesity & Hypoxia	[64,79,80]
Duration	Transient “Pulse”	Sustained, chronic stabilization	[68,70]
Metabolic Effect	Boosts glycolysis to fuel heat production	Blocks mitochondrial respiration; forces inefficient glycolysis (Warburg-like effect)	[64,68,70]
Mitochondrial Impact	Supports mitochondrial health and function	Suppresses mitochondrial biogenesis and fat burning	[6,81]
Physiological Outcome	Promotes healthy ‘browning’ of fat for thermogenesis	Drives adipose tissue dysfunction: fibrosis, inflammation, insulin resistance	[68,82]
Angiogenic Response	VEGF induction; improves perfusion	Impaired vascularization; ECM deposition	[83,84]
Systemic Consequence	Supports cold tolerance, increases EE	Promotes insulin resistance, obesity	[83,85]
Tumor–adipose crosstalk (peritumoral niche)	Not a canonical thermogenic program; context-dependent (host/systemic BAT activation may alter nutrient competition)	Tumor-driven chronic hypoxic/glycolytic pressure in adjacent adipose; ↑GLUTs and lactate axis; VEGF-linked angiogenic remodeling; “browning” markers may appear but can support tumor progression rather than net thermogenic benefit	[86,87,88,89]

**Table 2:** Stratified therapeutic interventions mapped onto RTF-Compass coordinates in adipose tissue.

Target State/Mechanism	Pharmacological Agent (Compass Vector)	Molecular Mechanism	Therapeutic Outcome	Reference
Iron Overload & Fenton Stress	Deferoxamine (DFO) or Ciclopirox (CPX)	Iron chelators; inhibitors of iron metabolism	Suppress ferroptosis by limiting Fenton chemistry and dampening BAT thermogenic activity through reduced iron-dependent redox signaling	[133,207]
Lipid Peroxidation	Ferrostatin-1, Liproxstatin-1	Lipid peroxidation inhibitors; radical-trapping antioxidants	Prevent ferroptotic membrane damage and mitigate accumulation of lipid hydroperoxides	[208,209,210]
GSH Depletion	Erastin	Inhibition of System xc− (cystine/glutamate antiporter)	Deplete glutathione (GSH) and inactivate GPX4 to enhance lipid peroxidation and promote ferroptosis	[30,175,211]
β3-Adrenergic Signaling	Mirabegron	β3-adrenergic receptor agonist	Improve BAT thermogenesis through β-AR stimulation and enhanced thermogenesis	[5,195,212,213]
PPARγ Signaling	Thiazolidinediones (TZDs) and INT131	PPARγ agonist/selective PPARγ modulator (SPPARM; partial agonist)	Improve insulin sensitivity and adipose function; may promote adipogenesis/weight gain; predicted benefit is context-dependent	[214,215,216]

## Data Availability

No new data were created or analyzed in this study. Data sharing is not applicable to this article.

## Abbreviations

The following abbreviations are used in this manuscript:

AATBC	Long noncoding RNA AATBC
ACSL4	Acyl-CoA synthetase long-chain family member 4
AREs	Antioxidant response elements
ATT	Adipose tissue thermogenesis
ATP2A2	ATPase sarcoplasmic/endoplasmic reticulum Ca^2+^ transporting 2 (SERCA2)
BAT	Brown adipose tissue
CPX	Ciclopirox
DFO	Deferoxamine
DiHETE	Dihydroxyeicosatetraenoic acid (e.g., 5,15-DiHETE)
FRC	Ferroptosis resistance capacity
FSI	Ferroptosis signaling index
FSP1	Ferroptosis suppressor protein 1
FTH	Ferritin heavy chain
GLUTs	Glucose transporters
GPX4	Glutathione peroxidase 4
GSH	Glutathione (reduced glutathione)
HIF-1α	Hypoxia-inducible factor 1 alpha
HIF-1β	Hypoxia-inducible factor 1 beta (ARNT)
IL-6	Interleukin 6
INT131	INT131 (selective PPARγ modulator; SPPARM)
KEAP1	Kelch-like ECH-associated protein 1
Lexis	Long noncoding RNA Lexis
LINC00473	Long intergenic non-protein coding RNA 473
LPCAT(s)	Lysophosphatidylcholine acyltransferase(s)
MCP-1	Monocyte chemoattractant protein 1
MDA	Malondialdehyde
NADPH	Nicotinamide adenine dinucleotide phosphate (reduced form)
Nfe2L1	Nuclear factor, erythroid 2 like 1
NRF2	Nuclear factor erythroid 2–related factor 2 (NFE2L2)
PDH	Pyruvate dehydrogenase
PDK	Pyruvate dehydrogenase kinase
PGC-1α	PPARγ coactivator 1 alpha
PGC-1β	PPARγ coactivator 1 beta
PHD	Prolyl hydroxylase domain enzyme
PHD2	Prolyl hydroxylase domain-containing protein 2
PPARγ	Peroxisome proliferator-activated receptor gamma
ROS	Reactive oxygen species
RTF-Compass	Redox–Thermogenesis–Ferroptosis Compass
TCA	Tricarboxylic acid
TfR1	Transferrin receptor 1
TFW	Thermogenic Ferroptosis Window
TNF-α	Tumor necrosis factor alpha
TZDs	Thiazolidinediones
UCP1	Uncoupling protein 1
VEGF	Vascular endothelial growth factor
WAT	White adipose tissue
β-AR	Beta-adrenergic receptor
β3-AR	Beta-3 adrenergic receptor
